# Analphoid supernumerary marker chromosome characterized by aCGH and FISH as inv dup(3)(q25.33qter) *de novo *in a child with dysmorphic features and streaky pigmentation: case report

**DOI:** 10.1186/1755-8166-1-19

**Published:** 2008-08-14

**Authors:** Sabita K Murthy, Ashok K Malhotra, Preenu S Jacob, Sehba Naveed, Eman EM Al-Rowaished, Sara Mani, Shabeer Padariyakam, R Pramathan, Ravi Nath, Mahmoud Taleb Al-Ali, Lihadh Al-Gazali

**Affiliations:** 1 Genetics Center, Department of Health and Medical Services, DHA, Dubai, UAE; 2 Department of Pediatrics, Faculty of Medicine and Health Sciences, Al-Ain Hospital, United Arab Emirates University, Al Ain, UAE

## Abstract

**Background:**

Small supernumerary marker chromosomes (sSMC) occur in 0.075% of unselected prenatal and in 0.044% of consecutively studied postnatal cases. Individuals with sSMC present with varying phenotype, ranging from normal to extremely mild or severe depending on the chromosomal region involved, the euchromatic content present and degree of mosaicism. Except for chromosomes 15 and 22, the number of reported cases of sSMC is extremely small to provide us with a good genotype-phenotype correlation. Analphoid sSMC are even rarer. To our knowledge only eight cases of analphoid inversion-duplication 3q sSMC are reported so far.

**Results:**

We describe here a one month old female child with several dysmorphic features and with a *de novo *analphoid supernumerary marker chromosome only in cultured skin fibroblast cells and not in lymphocytes. The marker was characterized as analphoid inversion-duplication 3q25.33-qter by oligo array comparative genomic hybridization (aCGH) and fluorescence in situ hybridization (FISH) studies. The final skin fibroblast karyotype was interpreted as 47,XX,+der(3).ish inv dup(3)(qter-q25.33::q25.33-qter)(subtel 3q+,subtel 3q+) *de novo*.

**Conclusion:**

In addition to the eight reported cases of analphoid inversion-duplication 3q supernumerary marker in the literature, this is yet another case of 3q sSMC with a new breakpoint at 3q25.33 and with varying phenotype as described in the case report. Identification of more and more similar cases of analphoid inversion-duplication 3q marker will help in establishing a better genotype-phenotype correlation. The study further demonstrates that aCGH in conjunction with routine cytogenetics and FISH is very useful in precisely identifying and characterizing a marker chromosome, and more importantly help in providing with an accurate genetic diagnosis and better counseling to the family.

## Background

Small supernumerary marker chromosomes occur in 0.075% of unselected prenatal cases and in 0.044% of consecutively studied postnatal cases, and majority of them are *de novo *in origin [1-4]. Phenotype of individuals with *de novo *sSMC vary from normal to extremely mild or severe, depending on the chromosomal region involved and the euchromatic content present [5-7]. Although a number of reports describe the occurrence of a variety of sSMC for nearly all the chromosomes, the number for each type is not large enough to suggest a good genotype-phenotype correlation for a given sSMC, except for inv dup(15) and inv dup(22) where the phenotypic consequences are well described [6,8-10]. We describe here the phenotype and corresponding molecular cytogenetic results of a child with dysmorphic features. This is yet another case of analphoid 3q supernumerary marker chromosome involving a new break point at 3q25.33. The marker is characterized as inversion-duplication 3q25.33-qter by oligo aCGH and FISH studies and it also reveals tissue specific mosaicism.

## Case presentation

A one month old female child presenting with several dysmorphic features was referred to us for cytogenetic studies. She was the second child of unrelated parents. The first child was normal. The pregnancy and delivery at term were normal. Birth weight was 2.2 Kg, length 45 cm and head circumference was 32 cm. At birth she was noted to have several dysmorphic features including: prominent hairy forehead with hair extending up to the cheeks, upslanting palpebral fissures, depressed nasal bridge with short nose and very smooth philtrum, thin upper lip which was turning downward, low set ears, micrognathia, chubby cheeks, contracture of the fingers with postaxial polydactyly of left hand, widely separated toes which were overlapping, streaky pigmentation on the inner aspect of both fore arms distributed along the lines of Blaschko. Opthalmological exam was normal. Echocardiography showed sub-aortic ventricular septal defect (VSD), pulmonary hypertention and moderate valvular pulmonary stenosis. Skeletal survey showed a tiny projection of the tip of coccyx, a tail-like sacrococcygeal appendage. Computed tomography scan of the lumbosacral spine showed prominence of coccyx and outward projection. Magnetic resonance imaging of the brain showed partial hypoplasia of the corpus callosum and slight hypoplasia of the cerebellum.

## Results

**Figure 1:**
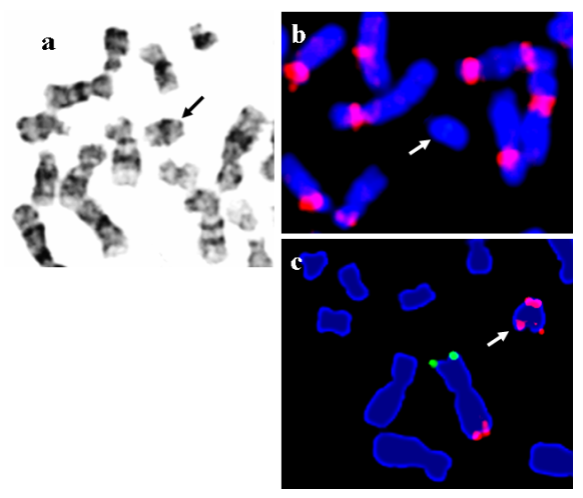
a) Partial G-banded metaphase from skin fibroblast cell showing the marker chromosome (arrow). b) Supernumerary marker chromosome showing no hybridization with human alpha-satellite Pan-centromeric probe (Cambio). (c) Chromosome 3 specific subtelomeric FISH (Mix 3 – Vysis) showing one normal chromosome 3 (3ptel green/3qtel red) and the marker chromosome with two red signals for 3q subtelomere, confirming the marker to be inversion-duplication 3q25.33-qter.

**Figure 2:**
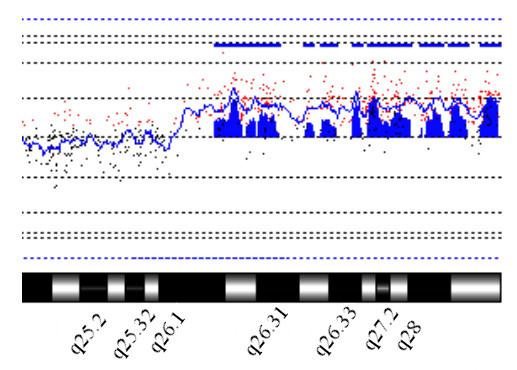
Oligo aCGH study using Agilent 44 K oligo array. Labeling and hybridization are as described in methods. Figure shows a partial cytogenetic profile of chromosome 3q and amplification of chromosomal region 3q25.33-qter.

Cytogenetic study by Giemsa banding (G-banding) showed normal 46,XX chromosomes in all the 50 lymphocyte cells and 47,XX,+mar in all the 70 skin fibroblast cells studied (Fig. 1a). The marker chromosome was constitutive heterochromatin band (C-band) and nucleolar organizer region (NOR) staining negative. Fluorescence in situ hybridization studies using Pan-centromeric alpha-satellite FISH probe (Cambio) showed no hybridization onto the marker chromosome (Fig. 1b), confirming it to be analphoid. Immuno-FISH studies for centromere specific proteins could not be undertaken to confirm the formation of a neocentromere. However, the fact that the marker was confirmed as analphoid by FISH and was also found to be highly stable in skin fibroblast culture, strongly suggest of a possible neocentromere formation. Peripheral blood chromosomes of the parents were normal by G-banding and by all chromosome specific sub-telomeric FISH analysis. Array-CGH studies on cultured skin fibroblast cells of the patient showed amplification of oligo-probes from probe FLJ14153 at 3q25.33 (160.03 Mb) to 3qter (199.29 Mb) (Fig. 2) confirming the origin and breakpoint of the marker chromosome. The observation was further validated by FISH studies using chromosome 3 specific subtelomere FISH probe (Vysis), which revealed the marker to be inversion-duplication 3q25.33-qter (Fig. 1c).

## Discussion

Supernumerary marker chromosomes are a cause of great concern and pose huge challenge in routine medical practice, particularly in genetic diagnosis and counseling. Precise identification and complete characterization of the marker chromosome is very important to understand the underlying cause of the disease and to establish a good genotype-phenotype correlation.

Analphoid markers are rare observations, in which the marker chromosome lacks centromeric alphoid DNA sequences, that are otherwise typically present in normal functional centromeres. Survival and stability of such an analhpoid marker chromosome depends on the formation of a neocentromere [11]. At present 73 cases of neocentromeric sSMC originating from different chromosomes are reported in the literature, of which 13q, 15q and 3q are the most frequent observations [1]. To our knowledge only 9 cases of analphoid 3q inversion-duplication marker (including the present case) are reported so far, involving chromosomal break points 3q22.3, 3q25.33, 3q26.2, 3q27.1, 3q27.2 and 3q28 [12-19]. A brief summary of the clinical features, chromosomal breakpoints and degree of mosaicism of the reported cases with 3q inversion-duplication supernumerary marker chromosome is presented in Table 1. As can be seen, the most frequent break point is at 3q26.2 (4 out of 9 cases) suggesting that this is possibly the most common site of neocentromere formation in a 3q marker. However, findings of other break points between 3q22 to 3q28 strongly suggest that this region probably has several potential hotspots of necentromere formation.

**Table 1:** Karyotype and clinical presentation of individuals reported with a supernumerary analphoid inversion-duplication 3q marker chromosome

Case no.	Karyotype	Clinical features	References
1	47,XY,+der(3) **inv dup(3)(qter-q22.3::q22.3-qter) **in dark skin fibroblast only (87%) 46,XY (in PBL & light skin)	Lumbosacral maningeocele, mental retardation, sparse hair, short limbs, hypoplasia of digital phalanges, agensis of nails and clinodactyly of fifth finger, ambiguous genitalia, depressed nasal bridge, anteverted nostrils lines of Blaschko, severe developmental delay.	[12,19]
2	47,XX,+der(3) **inv dup(3)(qter-q25.33::q25.33-qter) **in skin fibroblast only (100%) 46,XX (PBL)	Multiple congenital anomalies, prominent hairy forehead, low set ears, micrognathia, postaxial polydactyly of left hand, depressed nasal bridge, short nose, lines of Blaschko, sub arortic VSD, pulmonary hypertension, tail-like sacrococcygeal appendage, hypoplasia of corpus callosum.	present case
3	47,XY,+der(3) **inv dup(3)(qter-q26.2::q26.2-qter) **in skin fibroblast (57%)	Abortus with high arched palate, postnuchal edema, single transverse palmer crease on rt. hand lumbosacral myelomengiocele, Arnold-Chiari malformation, asymmetry of the kidneys, renal dysplasia.	[13,19]
4	47,XY,+der(3) **inv dup(3)(qter-q26.2::q26.2-qter) **in skin fibroblast (88%) in PBL (2.5%)	Enlargerd kidney, streaky hypopigmentation of skin, wide open anterior and posterior fontanel, rt preauricular pit, accessory nipples, postaxial polydactyly, clinodactyly of 5^th ^finger, rocker bottom feet, seizures, duplication of rt kidney, right pulmonary srtery stenosis, developmental delay.	[14,19]
5	47,XY,+der(3) **inv dup(3)(qter-q26.2::q26.2-qter) **in skin fibroblast 46,XY (PBL)	Mild developmental delay, attention-deficit hyperactivity, asymmetry of hands and legs, lines of irregular skin pigmentation consistent with the lines of Blaschko, macrocephaly.	[15,19]
6	47,XX,+der(3) **inv dup(3)(qter-q26.2::q26.2-qter) **in skin fibroblast (24%) 46,XX (PBL)	Skeletal abnormalities, limb stiffness, abnormal skin pigmentation, developmental delay.	[16,19]
7	47,XY,+der(3) **inv dup(3)(qter-q27.1::q27.1-qter) **in dark skin fibroblast (6%) in PBL (30%) 46,XY (100% in light skin)	22 year old man, normal intelligence, onset of pigmentary anomalies at age 10–12 years, lines of Blaschko, otherwise healthy and not dysmorphic.	[17,19]
8	47,XX,+der(3) **inv dup(3)(qter-q27.2::q27.2-qter) **in PBL (71%)	Swirly areas of hyperpigmentation, bilateral preauricular pits, hypotonia, developmental delay, seizures.	[14,19]
9	47,XX,+der(3) **inv dup(3)(qter-q28::q28-qter) **(100% in PBL)	Marked developmental delay, Hypognathia, atypical epicanthus, slight hirsutism, bilateral icthyosiform hyperkerotosis of palms and sole, hypotonia, hyporeflexia, cannot speak properly.	[18,19]

(PBL = peripheral blood lymphocyte culture)

Although all of the above cases with an analphoid 3q marker chromosome share some common clinical features, they still show widely varying phenotypes, probably due to the presence of varying euchromatic content as well as varying degree of mosiaicism (Table 1). Except for the case described by Portnoi et al [17] where the patient presented only with lines of Blaschko but otherwise healthy and not dysmorphic, all the other reported cases presented with developmental delay, severe dysmorphic features and multiple congenital anomalies involving several organs and systems. Our present case with break point at 3q25.33 presented with strikingly distinct phenotype as described in the case report. The typical facial appearance and the prominent hairy forehead are distinctive features, in addition to the cardiac and skeletal abnormalities that are similar to the cases described by Gimelli et al and Cockwell et al[12,13].

Mosaicism is reported in 59% of cases where the sSMC are found in association with a normal cell line [4]. Five of the nine cases discussed here showed tissue specific mosaicism where the 3q marker was present only in fibroblasts and not in lymphocytes (case number 1–3 and 5–6 of Table 1), other two cases showed varying degree of mosaicism in lymphocytes as well as in fibroblasts (case number 4 and 7 of Table 1), and the remaining two cases had the marker chromosome in 71% and 100% lymphocytes respectively (case number 8 and 9 of Table 1). Keeping in mind the specific clinical features of the patient and with the application of modern molecular cytogenetic detection methods such as aCGH and FISH, more and more similar cases are likely to be identified that would further help in obtaining a better genotype-phenotype correlation, and in providing with an accurate genetic diagnosis and better informed counseling, which is highly valuable to the patients.

## Methods

### Cytogenetic and fish studies

Cytogenetic study was carried out on peripheral blood lymphocytes by G-banding according to the standard procedures [20], and 50 G-banded metaphases were analyzed. FISH study was undertaken using all chromosome specific sub-telomere FISH probes (Vysis). Hybridization and washing was done as per manufacturer's protocol and 100 metaphases were analyzed. Due to the strong abnormal clinical presentation and presence of prominent streaky pigmentation, further studies were undertaken on cultured skin fibroblast cells by routine G-banding and 70 metaphase spreads were analyzed, followed by C-band and NOR staining [20]. To further characterize the marker chromosome, alpha-satellite Pan-centromeric (Cambio) and chromosome 3 specific sub-telomeric (Vysis) FISH studies were performed as per manufacturer's instructions.

### Oligo array CGH studies and validation

Oligo aCGH analysis was carried out using Human 44A microarray (Agilent) to determine the origin of the marker chromosome and to precisely identify the breakpoint region involved in the formation of the marker. The array contained *in situ *synthesized 60-mer oligonucleotides representing a total of 44,290 features. The oligonucleotide probes span the human genome with an average spatial resolution of approximately ~75 Kb, including coding and non-coding sequences, providing sufficient coverage for a genome-wide survey of DNA aberrations. Genomic DNA was extracted from cultured skin fibroblast cells of the patient by routine Proteinase K method and co-hybridized with normal male control DNA (Promega). DNA labelling, hybridization to oligonucleotide-array and post washing was carried out according to the protocol from Agilent Technologies and as described in Murthy et al [21]. Array CGH result was further validated by FISH study using chromosome 3 specific subtelomere FISH probe (Vysis).

## Competing interests

The authors declare that they have no competing interests.

## Authors' contributions

SKM conceived the study and drafted the manuscript. LG referred the patient, and RN and LG contributed clinical information. AKM, EEMR, SM, RP performed banding and cytogenetic analysis; SN and SP did FISH and PSJ did aCGH studies. MTA provided valuable support. All authors read and approved the final manuscript.

## Consent

This case report is presented with the consent of the patient's family.
